# 氧亲和力下降血红蛋白病Hb Sunshine Seth 1例报道并文献复习

**DOI:** 10.3760/cma.j.issn.0253-2727.2023.01.014

**Published:** 2023-01

**Authors:** 龙 常, 晨宇 王, 朝晖 李, 道斌 周, 欣欣 曹

**Affiliations:** 中国医学科学院、北京协和医学院北京协和医院血液内科，北京 100730 Department of Hematology, Peking Union Medical College Hospital, Chinese Academy of Medical Sciences & Peking Union Medical College, Beijing 100730 China

血红蛋白病Hb Sunshine Seth是一种编码珠蛋白α2链的基因（HBA2）突变造成血红蛋白携氧能力下降的血红蛋白病。该病首先报道于1979年，好发于高加索人群，多为常染色体显性遗传。临床表现为紫绀、血氧饱和度下降和无症状贫血[1]。实验室检查包括血气分析提示氧解离曲线右移，高效液相色谱（HPLC）、等电聚焦（IEF）或毛细管电泳（CE）可发现异常血红蛋白。本文我们报道血红蛋白病Hb Sunshine Seth 1例并总结复习相关文献，以提高对该病的认识。

## 病例资料

患者，男，39岁，因“体检发现血氧饱和度（SpO_2_）下降1个月”入院。20年前患者自觉口唇及四肢末端发绀，未诊治。1个月前体检SpO_2_ 80％~85％（自然状态）、90％（鼻导管吸氧3 L/min），活动量无改变，平素可慢跑5 km。外院查动脉血气分析：pH值7.41，氧分压（PaO_2_）91.2 mmHg（1 mmHg＝0.133 kPa），二氧化碳分压（PaCO_2_）39.8 mmHg，碳酸氢根浓度（HCO_3_^−^）25.6 mmol/L，氧饱和度（SaO_2_）80.1％，氧合血红蛋白（FO_2_Hb）78.5％。既往史：过敏性鼻炎、慢性荨麻疹、结肠息肉。个人史：长期吸烟、饮酒史。未接触亚硝酸盐。家族史：家族中多名亲属均口唇发绀。查体：血压：98/70 mmHg，脉率76次/min，SpO_2_ 84％（自然状态），口唇紫绀、肢端无青紫、心肺腹无殊、双下肢无水肿。

入院后查复查动脉血气见表1，血常规：WBC 4.73×10^9^/L、HGB 156 g/L、平均红细胞体积（MCV）92.1 fl、红细胞比容（Hct）46％、PLT 154×10^9^/L；肝肾功能、凝血、抗核抗体谱正常。外周血涂片正常。血红蛋白电泳：HbA 76.2％、HbA2 2.1％、异常血红蛋白HbX 21.7％。下肢超声、肺动脉CT血管造影（CTPA）未见血栓；心脏超声、胸部CT、肺功能、头部CT、头磁共振脑血管成像（MRA）、脑多普勒超声（TCD）未见明显异常。患者活动耐量正常，心肺功能未见异常，动脉血气分析示解离曲线右移，无其他继发影响因素。结合家族史，考虑异常血红蛋白病，氧结合力减低血红蛋白病可能性大。全外显子测序检出HBA2基因第2外显子杂合突变（c.283G>C、p.Asp94His）（图1），明确诊断氧亲和力下降血红蛋白病Hb Sunshine Seth。经家系调查，患者父亲与姐姐均检测到同样突变（图2）。

**表1:** 患者不同状态动脉血气分析结果（血气分析仪器：RADIOMETER ABL90）

状态	pH	PaCO_2_（mmHg）	PaO_2_（mmHg）	HCO_3_^−^（mmol/L）	SaO_2_（％）	FO_2_Hb（％）	FHHb（％）	p50（mmHg）
静息	7.4	44.4	89	26	79.2	78.2	20.5	58
运动后	7.3	38.6	106	19	80.6	79.3	19.1	68
吸氧后	7.3	35.4	241	19	89.3	87.8	10.5	133

注 PaCO_2_：二氧化碳分压；PaO_2_：氧分压；HCO_3_^−^：碳酸氢根浓度；SaO_2_：动脉血氧饱和度；FO_2_Hb：氧合血红蛋白浓度；FHHb：脱氧血红蛋白浓度；p50：血红蛋白50％氧合时的氧分压

**图1:**
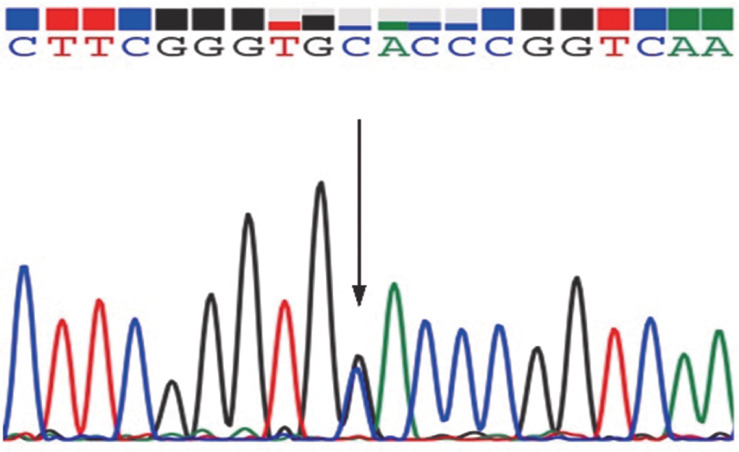
血红蛋白病Hb Sunshine Seth患者全外显子测序结果（箭头所示为突变位点）

**图2:**
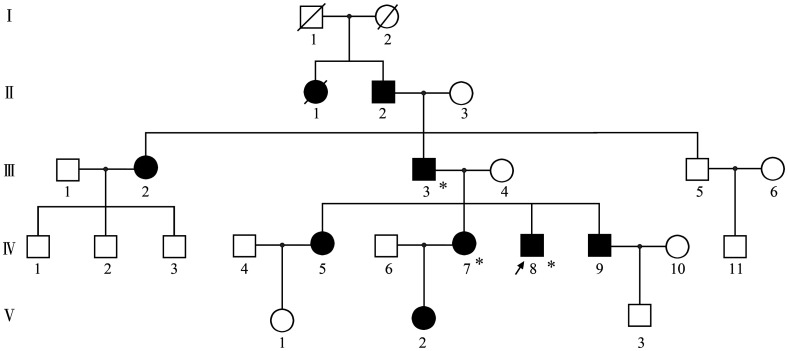
血红蛋白病Hb Sunshine Seth患者家系图 注 ↗：先证者；■、●：有临床表现（紫绀、血氧下降）男性、女性；□、○：无临床表现男性、女性；*：基因检测阳性；/：死亡

## 讨论及文献复习

人类血红蛋白是由两对（4条）血红蛋白单体聚合而成的四聚体，每个单体由珠蛋白和血红素构成。每个珠蛋白分子有两对肽链，一对是α链一对是非α链（β、γ、δ、ε等）。正常人出生后有三种血红蛋白：血红蛋白A（HbA，α2β2链）、血红蛋白A2（HbA2，α2δ2链）、胎儿血红蛋白（HbF，α2γ2链）[2]。

每个血红蛋白分子以两种不同的物理形式存：易与氧结合的疏松形态（R型）和易与氧解离的紧密形态（T型），从T型到R型的转变意味着血红蛋白可以结合更多的氧[3]。

血红蛋白病是一类由于血红蛋白分子的珠蛋白肽链结构异常引起的疾病。T型构象血红蛋白分子中，四条多肽链之间由8对盐键共同维持紧密连接的状态。其中珠蛋白α1链94位天冬氨酸（Asp）残基和β2链102位精氨酸（Arg）残基之间形成的盐键是氧结合与解离的关键位点，可以稳定T型和R型的结构[4]。编码α1链94位和β2链102位氨基酸的碱基序列异常可影响血红蛋白氧亲和力。如Hb Bassett、Hb Setif、Hb Capa、Hb Titusville、Hb Roanne、Hb Kirksey等疾病均有珠蛋白α1链第94对密码子突变，造成血红蛋白多肽链之间的盐键在氧合过程中不易被破坏，T型更难以转变为R型，导致氧亲和力下降。这类疾病多发生在欧美人群，临床表现为紫绀或仅查体发现血氧饱和度下降，伴或不伴轻度贫血，血红蛋白电泳发现异常血红蛋白，与本例临床表现相似，既往文献报道和病例特点与突变位点见表2[5]–[11]。而同样在 β2链102Arg突变的疾病中，Hb Beth Irrael（β102 Arg→Ser）、Hb Richmond（β102 Arg→Lys）造成血红蛋白氧亲和力增高，Hb Kansas（β102 Arg→Thr）和Hb Saint Mande（β102 Arg→Tyr）造成血红蛋白氧亲和力降低[12]–[15]。截止目前，已发现有近100种氧亲和力增高血红蛋白病和近70种氧亲和力降低血红蛋白病，目前已知血红蛋白病类型可在血红蛋白病数据库（https://globin.bx.psu.edu/hbvar/menu.html）查阅。

**表2:** 氧亲和力下降血红蛋白病的临床表现和突变位点

参考文献	血红蛋白病种类	氨基酸改变	碱基改变	性别	年龄（岁）	国家/地区	临床表现	SpO_2_（％）	HGB（g/L）	MCV（fl）	Hb X（％）
本文	Hb Sunshine Seth	α94 Asp→His	GAC→CAC	男	39	汉族	紫绀	85	156	92.1	21.70
Heidenreich等[5]	Hb Sunshine Seth	α94 Asp→His	GAC→CAC	男	17	NM	无	85	98	86	16.10
Abdulmalik等[6]	Hb Bassett	α94 Asp→Ala	GAC→GCC	男	6	高加索	紫绀	90	119	NM	30
Wajcman等[7]	Hb Setif	α94 Asp→Tyr	GAC→TAC	男	73	马耳他	无	99	123	83.9	16
Dinçol等[8]	Hb Capa	α94 Asp→Gly	GAC→GGC	女	28	土耳其	无	NM	123	85	16.20
Kister等[9]	Hb Roanne	α94 Asp→Glu	GAC→GAG	女	73	法国	溶血	NM	118	NM	18
Schneider等[10]	Hb Titusville	α94 Asp→Asn	GAC→AAC	女	56	北欧	紫绀	83	133	91	15.60
Yudin等[11]	Hb Kirksey	α94 Asp→Val	GAC→GTC	男	37	加拿大	无	90	163	106	NM

注 SpO_2_：血氧饱和度；MCV：平均红细胞体积；NM：未提及

氧亲和力下降的血红蛋白病临床可仅表现为SpO_2_下降，伴或不伴紫绀。SpO_2_检测仪是根据氧合血红蛋白和去氧血红蛋白不同吸光度的比例进行计算，在体内存在异常血红蛋白的情况下会出现误差[16]。因此判断患者氧饱和度真实情况需要检测动脉血气分析中的PaO_2_和SpO_2_。血红蛋白携氧能力下降，所以p50（血红蛋白50％氧合时的氧分压）升高，氧解离曲线右移。虽然动脉SpO_2_下降，但实际上氧更容易在组织中解离，组织供氧反而增多，红血蛋白水平可能会代偿性下降，临床上可能会表现为轻度无症状性贫血[17]。在动物实验中已经证实，血红蛋白携氧能力下降能增加组织供氧、改善心功能不全，提高活动耐量等[18]。临床中遇到无法解释的低氧血症需要考虑血红蛋白病，尤其是存在SpO_2_与氧分压不匹配、氧解离曲线偏移等表现时[19]。常规实验室检查无法诊断血红蛋白病，需要进一步通过HPLC或电泳等方法发现异常血红蛋白，明确诊断需要基因检测。早期诊断氧亲和力下降的红血蛋白病可以避免临床中过度检查和治疗。

综上所述，临床医师应该提高对氧亲和力异常血红蛋白病的认识，对于临床中不明原因的无症状低氧血症的患者，需要考虑氧亲和力下降血红蛋白病。
